# Development of a *pyrG* Mutant of *Aspergillus oryzae* Strain S1 as a Host for the Production of Heterologous Proteins

**DOI:** 10.1155/2013/634317

**Published:** 2013-11-30

**Authors:** Selina Oh Siew Ling, Reginald Storms, Yun Zheng, Mohd Rohaizad Mohd Rodzi, Nor Muhammad Mahadi, Rosli Md Illias, Abdul Munir Abdul Murad, Farah Diba Abu Bakar

**Affiliations:** ^1^School of Biosciences and Biotechnology, Faculty of Science and Technology, Universiti Kebangsaan Malaysia, 43600 Bangi, Selangor, Malaysia; ^2^Centre for Structural and Functional Genomics, Concordia University, 7141 Sherbrooke Street West, Montréal, Québec, Canada H4B 1R6; ^3^Department of Biology, Concordia University, 7141 Sherbrooke Street West, Montréal, Québec, Canada H4B 1R6; ^4^Malaysia Genome Institute, Jalan Bangi, 43000 Kajang, Selangor, Malaysia; ^5^Department of Bioprocess Engineering, Faculty of Chemical Engineering, Universiti Teknologi Malaysia, 81310 Skudai, Johor, Malaysia

## Abstract

The ease with which auxotrophic strains and genes that complement them can be manipulated, as well as the stability of auxotrophic selection systems, are amongst the advantages of using auxotrophic markers to produce heterologous proteins. Most auxotrophic markers in *Aspergillus oryzae* originate from chemical or physical mutagenesis that may yield undesirable mutations along with the mutation of interest. An auxotrophic *A. oryzae* strain S1 was generated by deleting the orotidine-5′-monophosphate decarboxylase gene (*pyrG*) by targeted gene replacement. The uridine requirement of the resulting strain GR6 *pyrGΔ0* was complemented by plasmids carrying a *pyrG* gene from either *Aspergillus nidulans* or *A. oryzae*. **β**-Galactosidase expression by strain GR6 *pyrGΔ0* transformed with an *A. niger* plasmid encoding a heterologous **β**-galactosidase was at least 150 times more than that obtained with the untransformed strain. Targeted gene replacement is thus an efficient way of developing auxotrophic mutants in *A. oryzae* and the auxotrophic strain GR6 *pyrGΔ0* facilitated the production of a heterologous protein in this fungus.

## 1. Introduction

In Japan, the filamentous fungus *Aspergillus oryzae* is extensively used in the production of miso (soybean paste), sake (rice wine), and shoyu (soy sauce). Due to its proven safety record in the food industry, *A. oryzae* has been assigned the status Generally Recognized As Safe (GRAS) by the United States Food and Drug Administration (USFDA) and the World Health Organization (WHO) [1]. *A. oryzae* is also used for the production of homologous and heterologous proteins and metabolites [2]. The *A. oryzae* genome consists of eight chromosomes totalling 37 megabases (Mb) predicted to harbour 12,074 protein coding genes [3].

The *pyrG* gene encodes orotidine-5′-monophosphate (OMP) decarboxylase, an enzyme of the pyrimidine biosynthesis pathway that is important for uridine synthesis. The mutagen 5-fluoroorotic acid (5-FOA) inhibits cell proliferation in most organisms by entering the pyrimidine synthesis pathway where it is converted into the pyrimidine analogue fluoroorotidine monophosphate. Through the subsequent action of ribonucleotide reductase, fluoroorotidine monophosphate is converted into fluorodeoxyuridine, a potent suicidal inhibitor of the enzyme thymidylate synthase, which converts deoxyuridine monophosphate into the essential DNA precursor thymidylate [4]. Resistance to 5-FOA can be achieved by blocking the pyrimidine pathway through mutational inactivation of orotidine-5′-monophosphate (OMP) decarboxylase which catalyzes the decarboxylation of orotidine monophosphate (OMP) to form uridine monophosphate (UMP). In filamentous fungi, pyrimidine auxotrophs obtained through inactivation of the OMP decarboxylase gene (usually designated *pyrG*) are auxotrophic for uridine and/or uracil and are resistant to 5-FOA [5, 6]. The generation of 5-FOA-resistant *pyrG *mutants has been reported in other fungi including *A. aculeatus*, *A. niger, Sclerotinia sclerotiorum,* and *Trichoderma reesei* [5, 7–9]. Most *pyrG* mutants were isolated following either ultraviolet (UV) or chemical mutagenesis. Mutations induced in these ways are often unstable (i.e., they revert to the wild type) and may include multiple, undesirable mutations. In contrast, mutants generated by targeted gene replacement are stable as the targeted gene is removed from the genome. It is, however, more difficult to mutate multiple genes this way [10, 11].

A BLAST search using the *pyrG* sequence of *A. oryzae* S1 against the *A. oryzae *RIB40 genome database at the National Institute of Technology and Evaluation (NITE; http://www.bio.nite.go.jp/dogan) showed that *pyrG* is present as a single copy on chromosome 7 (contig SC011, location 2219667…2220565).

In this study, a gene replacement cassette was constructed consisting of the native* pyrG *locus extending 1.5 to 2.0 kb beyond the upstream and downstream termini of the *pyrG *coding region but lacking the open reading frame of the gene. Transformation of *A. oryzae* strain S1 with the gene replacement cassette generated 5-FOA-resistant uracil and uridine auxotrophs. Putative *pyrGΔ0* mutants were verified by comparing their growth on selective and nonselective media, PCR analyses of the *pyrG *region in the putative mutants and complementation with plasmids containing wild-type homologous and heterologous *pyrG* genes. Suitability of the *pyrGΔ0* mutant as a host for heterologous protein production was assessed using strain GR6 harbouring a plasmid in which the *pyrG* and heterologous *β*-galactosidase genes were integrated.

## 2. Materials and Methods

### 2.1. Fungus, Cultivation, and Plasmids

**Figure 1:**
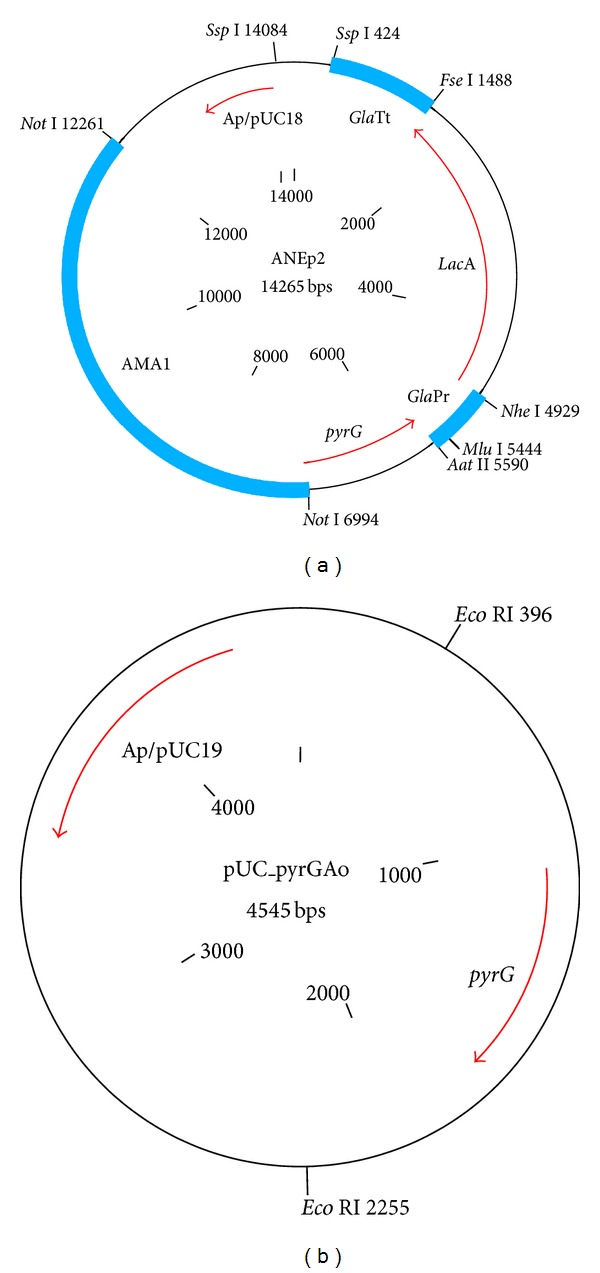
Plasmids used for transformation. (a) Plasmid ANEp2 containing the *pyrG* gene of *A. nidulans* and a *Gla*Pr-*lacA*-*Gla*Tt chimera (b) plasmid pUC_pyrGAo containing *pyrG* (1,839 bp) of *A. oryzae* strain S1.

*A. oryzae* strain S1was obtained from the Fungal Stock Culture Collection of the Molecular Mycology Laboratory, School of Biosciences and Biotechnology, Faculty of Science and Technology, Universiti Kebangsaan Malaysia. The fungus was maintained on a Potato Dextrose Agar (PDA; Difco, France) at 30°C and subcultured monthly. Fungal cultivation was carried out by inoculating onto a fresh PDA plate. Cultures were incubated for 7 days at 30°C. Plasmid ANEp2 (Figure 1(a)) is a shuttle vector capable of autonomous replication in Aspergilla [12]. In addition to the pUC18 backbone ANEp2 carries the *A. nidulans pyrG* gene as a selectable marker, an expression cassette (*Gla*Pr-*lacA*-*Gla*Tt) that expresses an *A. niger *secreted *β*-galactosidase, and the AMA1 sequence that supports autonomous replication in Aspergilli. ANEp2 was used to functionally test for complementation of uridine auxotrophy and reporter gene expression in an *A. oryzae pyrG* mutant. Plasmid pUC_pyrGAo containing the *pyrG* of the *A. oryzae* strain S1 was also used for complementation of uridine auxotrophy (Figure 1(b)) [13]. *Escherichia coli* strain DH5*α* was used for DNA manipulation.

### 2.2. Media

Polypeptone dextrin (PD) medium containing 1.0% (w/v) polypeptone, 2.0% (w/v) glucose, 0.5% (w/v) KH_2_PO_4_, 0.1% (w/v) NaNO_3_, and 0.05% (w/v) MgSO_4_·7H_2_O was used for liquid cultivation of *A. oryzae* [14]. Czapek-Dox (CD) medium composed of 0.2% (w/v) NaNO_3_, 0.1% (w/v) K_2_HPO_4_, 0.05% (w/v) MgSO_4_·7H_2_O, 0.05% (w/v) KCl, 0.001% (w/v) FeSO_4_ and 3.0% (w/v) sucrose at pH 5.5 (supplemented with 0.2% (w/v) uracil and 20 mM uridine) was used for spheroplast cell wall regeneration. Spheroplasts were overlaid with CD medium containing 0.5% (w/v) agar and 5.0% (w/v) NaCl onto CD medium containing 2.0% (w/v) agar and 5.0% (w/v) NaCl [15]. Cultivation and selection of positive transformants were performed using CD minimal agar media [16] composed of 0.2% (w/v) NaNO_3_, 0.1% (w/v) KH_2_PO_4_, 0.05% (w/v) MgSO_4_·7H_2_O, 0.05% (w/v) KCl, 0.002% (w/v) FeSO_4_·7H_2_O, 2.0% (w/v) glucose, 0.05% (w/v) NaCl, and 2% (w/v) agar at pH 5.5 containing 2 mg/mL 5-FOA, 0.2% (w/v) uracil and 20 mM uridine.

### 2.3. Replacement Cassette Construction

**Figure 2:**
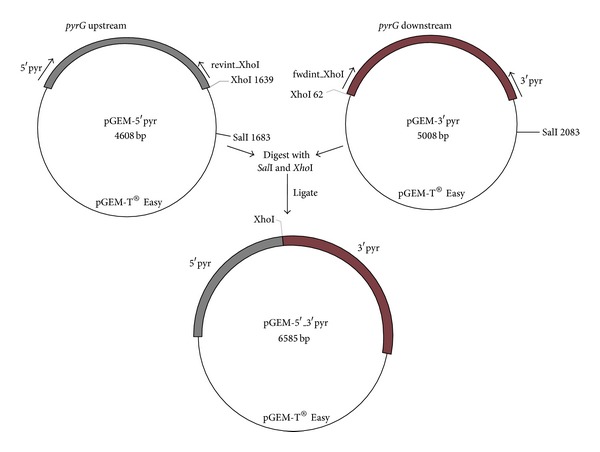
Schematic diagram outlining assembly of the *pyrG* gene replacement cassette.

The cassette used to delete the *A. oryzae pyrG* (Figure 2) was assembled as follows: genomic DNA of *A. oryzae* strain S1 was used as the PCR template to amplify the 1.6 kb and 2.0 kb regions upstream and downstream of the *pyrG *coding region, respectively. The upstream region was amplified with the primers 5′pyr and revint-XhoI while the downstream region was amplified using the primers fwdint-XhoI and 3′pyr (Table 1). Both PCR products were ligated into the vector, pGEM-T Easy (Promega, Fitchburg, WI, USA). Plasmids pGEM-5′pyr and pGEM-3′pyr were identified by restriction endonuclease digestion and verified by sequencing. Finally, the two regions flanking the *pyrG* coding region were assembled into the *pyrG *replacement cassette by ligating the plasmids pGEM-5′pyr and pGEM-3′pyr digested with *Xho*I and *Sal*I. Restriction endonuclease mapping and sequencing were used to verify the construction of pGEM-5′_3′pyr.

### 2.4. Spheroplast Preparation and Transformation

Spheroplasts were produced based on the methods of Ushijima et al. [14] and Liew [17] as follows: *A. oryzae* conidia (10^7^ conidia/mL) were cultivated in 50 mL PD at 30°C for 20 h. Mycelia (3 g wet weight) were pretreated with DTT-lysis buffer containing 10 mM dithiothreitol (DTT) and 10 mM Tris-HCl for 1 h at 30°C by shaking at 75 rpm prior to incubation with 70 mg/mL lysing enzyme (*Trichoderma harzianum*, Sigma-Aldrich Corp. St. Louis, MO, USA) solution containing osmotic buffer (0.8 M MgSO_4_ in 0.01 M phosphate buffer) for 4 h at 30°C (agitation—75 rpm). The mixture containing spheroplasts was transferred to a 50 mL centrifuge tube (Greiner Bio-One, UK) and 0.6 M sorbitol was added slowly to the mixture. The mixture was spun at 4,000 rpm, 4°C for 30 min. The interphase layer between the lysis buffer and sorbitol was collected and transferred to a new 50 mL tube with the addition of STC buffer (1.2 M sorbitol, 0.01 M Tris-HCl, 0.01 M CaCl_2_). Transformation was carried out according to Storms et al. [12] and Wernars et al. [18] using 1.6 × 10^6^ viable spheroplasts in STC mixed with 10 *μ*g purified PCR product (3.6 kb amplified DNA fragment consisting of the 5′pyr and 3′pyr regions of the *pyrG* gene replacement cassette) and 1.2 M sorbitol as the osmotic stabilizer. Subsequently, 0.5 mL aliquots containing 8 × 10^5^ transformed spheroplasts were mixed into 0.5% CD overlay agar, poured onto 2% CD agar plates supplemented with 0.2% uracil and 20 mM uridine, and incubated at 30°C for 4–6 days.

### 2.5. Screening and Analysis of Transformants

5-FOA resistant colonies were tested for auxotrophy by patching on CD medium and CD medium with uracil and uridine. Verification that the 5-FOA-resistant auxotrophs arose via deletion of the *pyrG* coding region was done by PCR analysis of total DNA using primers Pyr5′-out and Pyr3′-out (Table 1) designed to be complementary to the known genomic (obtained from the *A. oryzae *RIB40 NITE genome database) sequence just beyond the upstream and downstream boundaries of the replacement cassette. The *pyrGΔ0* mutants were also tested by complementation with plasmids pUC_pyrGAo and ANEp2 harbouring *A. oryzae *and *A. niger pyrG,* respectively.

### 2.6. *β*-Galactosidase Expression

Levels of *β*-Galactosidase activity expressed by *A. oryzae *strain GR6* pyrGΔ0* and GR6* pyrGΔ0* transformants harbouring ANEp2 were determined using filtered medium from 7-day old static cultures. Static cultures were generated by inoculating 1.0 × 10^6^ conidiospores into Petri dishes containing 25 mL of MMJ medium as described by Käfer [19] using 15% maltose as the carbon source (along with the necessary supplements) [20], at 30°C. *β*-Galactosidase assays were performed by adding culture filtrates to a reaction-buffer containing 50 mM sodium acetate (pH 4.1) and 4 mg/mL of orthonitrophenyl-*β*-D-galactoside (ONPG), and incubating for 10 min at 37°C. Assays were stopped by adding 1 volume of 1 M sodium carbonate and the absorbance was read at 415 nm. *β*-Galactosidase activity is expressed in units (U) per mL of culture, where one unit is the amount of enzyme needed to hydrolyze 1 *μ*moL of ONPG to orthonitrophenol per min at 37°C. Western blot analyses were carried out using 20 *μ*L samples of concentrated culture filtrate using ultra-filtration spin columns with a molecular weight cut-off of 10 kDa (“Vivaspin,” Sartorius, Göttingen, Germany) and fractionated by 10% SDS-PAGE. Fractionated proteins were transferred to nitrocellulose membranes and visualized using a rabbit anti-lac A antiserum, an anti-rabbit IgG labelled with horseradish peroxidase (Promega) and a chemiluminescent detection kit (Thermo Fisher Scientific, Waltham, MA, USA). Rabbit serum containing polyclonal antibodies directed against *A. niger β*-galactosidase was generated as follows. *A. niger β*-galactosidase was expressed and purified by ion exchange chromatography. Purified *β*-galactosidase (200 *μ*g with complete Freund's Adjuvant for immunization) was introduced by subcutaneous injections into New Zealand white rabbits. The initial injection was followed by two booster injections (100 *μ*g purified *β*-galactosidase with Incomplete Freund's Adjuvant). For serum preparation, the collected blood was stored at 4°C overnight and centrifuged for 30 min at 4,000 rpm. The collected supernatant was stored at −20°C. Enzyme-linked immunosorbent assay (ELISA) was used for the evaluation of antibody positive titer in the rabbit serum.

## 3. Results

### 3.1. Deletion of the *A. oryzae* Strain S1 *pyrG*

**Figure 3:**
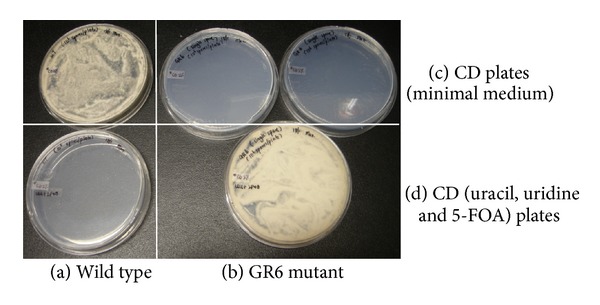
Media plates showing growth or no growth of the *A. oryzae* S1 wild type (column (a)) and GR6 *pyrG* mutant (column (b)). Column (a), ability of the wild type strain S1 (row (c)) and Column (b), the inability of the GR6 *pyrG* mutant strain (row (c)) to grow on minimal medium CD plates. Column (a), inability of the wild type strain S1 (row (d)) and Column (b), ability of the GR6 *pyrG* mutant strain (row (d)) to grow on minimal medium CD plates supplemented with uracil, uridine and 5-FOA.

**Figure 4:**
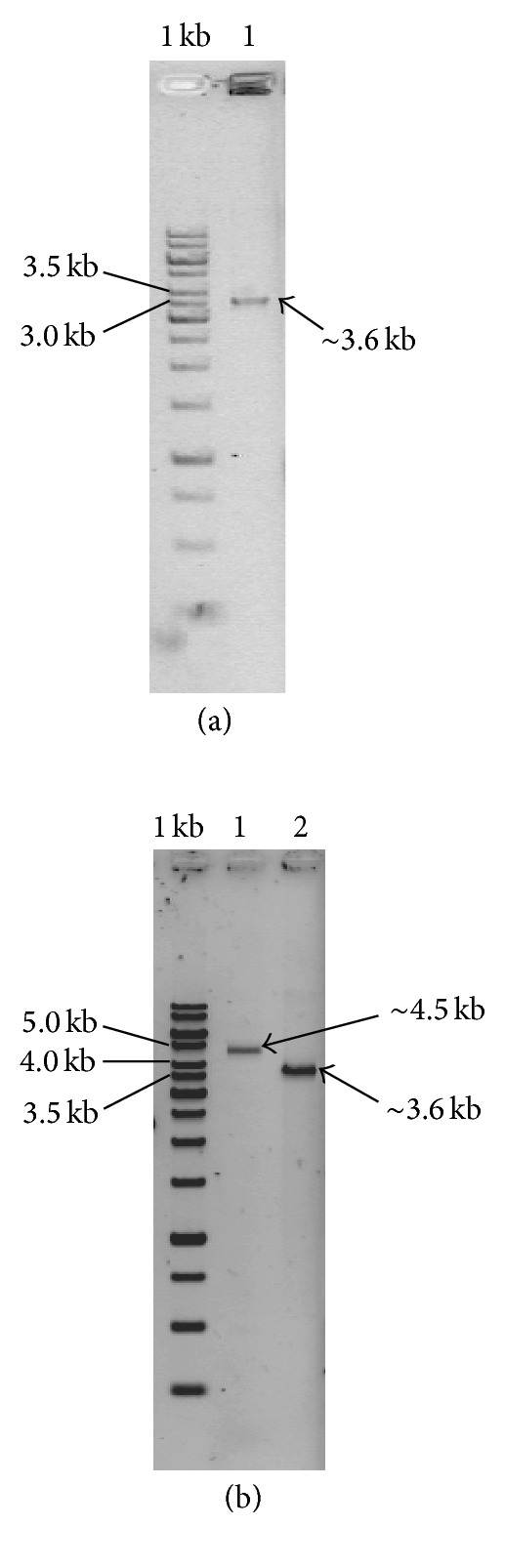
Gel electrophoretic profile of the (a) (lane 1) gene replacement cassette consisting of the 5′ and 3′*pyrG* DNA inserts (without the coding region) with the size of ~3.6 kb and (b) genomic PCR product of the *A. oryzae* S1 wild type strain (lane 1) and its mutant derivative *pyrGΔ0*, strain GR6 (lane 2). Lane M: 1 kb DNA ladder (Fermentas, Canada).

The 3.6 kb *pyrGΔ0* gene replacement cassette was amplified using primers 5′pyr, and 3′pyr, and plasmid pGEM-5′_3′pyr as the template (Figure 4(a)). Generation of a *pyrGΔ0* derivative of *A. oryzae *was performed by transforming strain S1 with the 3.6 kb *pyrGΔ0 *cassette. Initial attempts to regenerate transformed spheroplasts were performed on CD plates supplemented with uracil, uridine, and 5-FOA. This strategy, which selects for *pyrGΔ0* gene replacements immediately after transformation of spheroplasts with the PCR product, is similar to the approach used to isolate *pyrG *mutants of *Trichoderma reesei* (irradiated spores are platedout on minimal medium containing uridine and 5-FOA—Long et al. [5]). However, colonies derived from the experiments described here with *pyrG* failed to yield FOA resistant colonies. Perhaps 5-FOA-resistant *pyrGΔ0* mutants were not obtained because multiple nuclei were present in the spheroplasts, and therefore, a single *pyrGΔ0* locus could not prevent production of the suicide inhibitor FdUMP. To address this possibility, the transformation protocol was modified by allowing nuclei in which the *pyrG *gene was deleted to segregate from wild-type nuclei by plating the transformed spheroplasts (about 8 × 10^5^ per plate) onto nonselective regeneration plates containing uracil/uridine. Once a lawn of conidia was produced, the conidia were collected, diluted and plated at 8 × 10^6^ spores per plate on CD selective medium with 5-FOA, uracil, and uridine. Ten randomly selected colonies were chosen from over 100 colonies that formed and purified to homogeneity by two rounds of plating on CD uracil and uridine plates. Conidia prepared using the purified colonies were harvested and plated onto CD plates without uracil and uridine (10^6^ conidia per plate). Only one of the putative mutants, designated GR6 (Gene Replacement mutant number 6) did not grow and was therefore assumed to be completely stable (Figure 3).

### 3.2. Verifying that GR6 Harbours a *pyrGΔ0* Locus

PCR amplifications with primers Pyr5′-out and Pyr3′-out using the genomic DNA of strain S1 and strain GR6 as the templates, showed that a 0.9 kb deletion was introduced into the *pyrG* region of strain GR6 (Figure 4(b)), which is consistent with generation of the desired *pyrGΔ0* locus by homologous recombination.

Then, the *pyrG* auxotroph GR6 was transformed with plasmids pUC_pyrGAo and ANEp2. These plasmids contain the wild-type *pyrG* from *A. oryzae *(in pUC19) and *A. nidulans,* respectively. Both plasmids were introduced into and restored uracil and uridine prototrophy of GR6 with frequencies of three transformants per *μ*g of plasmid DNA for pUC_pyrGAo and 50 transformants per *μ*g of plasmid DNA for ANEp2. Furthermore, the frequency ratio of *pyrG *prototrophs obtained when transformation was performed with the pUC_pyrGAo versus the AMA plasmid was about 0.1. This is in accordance with the range of prototrophy frequency ratios obtained with integrating versus autonomously replicating plasmids previously reported with other fungal species [12, 21].

### 3.3. Protein Expression

**Figure 5:**
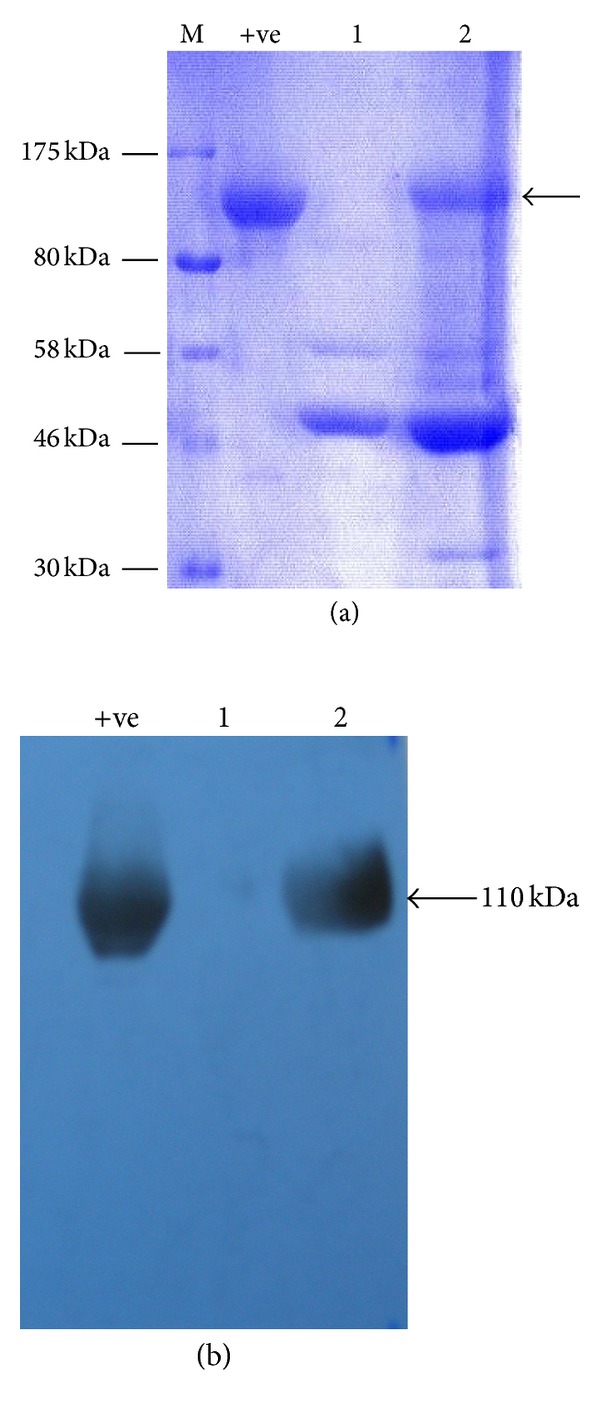
Analysis of GR6 culture filtrates. Panel (a): lane M, SDS-PAGE profile of prestained protein marker (New England Biolabs, USA); lane +ve, purified *A. niger β*-galactosidase isolated from a culture filtrate of *A. niger* transformed with the *Gla*Pr-*lacA*-*Gla*Tt expression cassette; lane 1, culture filtrate prepared using *A. oryzae* strain GR6 *pyrGΔ0*; and lane 2, culture filtrate prepared using GR6 *pyrGΔ0* transformed with ANEp2. Panel (b); Western blot performed by probing the gel shown in Panel (a) with polyclonal rabbit antibodies directed against *A. niger β*-galactosidase.

Total secreted proteins in culture filtrates of the untransformed strain GR6* pyrGΔ0* and GR6* pyrGΔ0* transformed with ANEp2 were analyzed by SDS-PAGE (Figure 5(a)) and Western blotting (Figure 5(b)). SDS-PAGE showed a very prominent 110 kDa protein, which is about the size expected of the *A. niger β*-galactosidase (Swiss-Prot accession number P29853) (UnitProtKB; http://www.uniprot.org/uniprot/P29853) [22] in the culture filtrate of ANEp2 GR6* pyrGΔ0* which was absent in the untransformed strain GR6* pyrGΔ0*. Western blot analyses confirmed that the major band was the *A. niger β*-galactosidase. As expected from the above, culture filtrates of the ANEp2 GR6* pyrGΔ0* transformants possessed high levels of *β*-galactosidase activity (135 U/mL), as compared to the untransformed strain GR6* pyrGΔ0* which expressed *β*-galactosidase at 0.80 U/mL.

## 4. Discussion

Replacement of a gene requires homologous recombination of the introduced DNA with the genome: gene replacement is achieved by a precise double crossover event [23]. We first sought these events by selecting for FOA-resistant clones. FOA inhibits the growth of the fungus but permits multiplication of cells deficient in OMP decarboxylase and because of this, are auxotrophic for uridine. Initially, we selected for *pyrGΔ0* gene replacements immediately after transfection using a similar plating procedure as has been used to isolate *pyrG *mutants of *T. reesei*, *A. aculeatus,* and* Penicillium nalgiovense* from irradiated spores [5, 7, 21]. In our hands, this technique failed to yield FOA resistant colonies. This was probably because multiple nuclei were present in each spheroplast, and therefore a single *pyrGΔ0* locus could not prevent production of the suicide inhibitor FdUMP. To test this, the transformation protocol was modified to allow cells containing putative *pyrG *deletions to segregate from wild-type ones by plating the transformed spheroplasts onto nonselective regeneration plates containing uracil and uridine. Then, the conidia from the nonselective plates were plated-out on CD selective medium containing 5-FOA, uracil and uridine. This led to isolation of the mutant strain, GR6 *pyrGΔ0*. PCR based tests confirmed that the *pyrG* region of strain GR6 was deleted.

It was possible to complement the uridine requirement of GR6 *pyrGΔ0* by introducing the plasmids pUC_pyrGAo and ANEp2 carrying *A. oryzae* or *A. nidulans pyrG*, respectively. This suggests that the AMA1 region of ANEp2, which supports autonomous plasmid replication in other fungi, also supports autonomous replication in *A. oryzae. *Furthermore, the transformation frequency of ANEp2 was about 10-fold greater than that obtained with the integrative plasmid pUC_pyrGAo. Transformation frequency ratios obtained with pUC_pyrGAo as compared to plasmids harbouring AMA1 were in accordance with those obtained with integrating versus autonomously replicating plasmids [12]. Also, Fierro et al. [21] transformed *P. nalgiovense* with integrative versus autoreplicative plasmids and found 60-fold higher frequencies for AMA1-based autonomously replicating plasmids compared with integrative plasmids. Autonomously replicating plasmids carrying the AMA1 region from *A. nidulans* transformed *P. nalgiovense* very efficiently and these plasmids also showed mitotic stability with low degrees of reorganization.

*β*-Galactosidase expression showed that the *A. oryzae* strain GR6* pyrGΔ0* was able to efficiently produce heterologous secreted proteins using the *A. niger* transcriptional and translational controls introduced via ANEp2. The strain GR6 *pyrGΔ0* transformed with ANEp2 containing *lacA* encoding *A. niger β*-galactosidase was more than 150 times higher than that obtained with the untransformed strain. The finding of this study is promising and this paves the way for the application of *A. oryzae* strain GR6* pyrGΔ0* in heterologous protein production given that this strain is amenable to genetic manipulation and that *A. oryzae* is widely used in biotechnology and the food industry.

## 5. Conclusion

Mutant strain GR6, a *pyrGΔ0* derivative of the *A. oryzae *strain S1, was constructed using targeted gene replacement. The *pyrG* auxotrophs were verified by assessing their ability to grow on selective and nonselective media and PCR analysis. Plasmids pUC_pyrGAo and ANEp2, harbouring the *pyrG* genes from *A. oryzae* and *A. nidulans*, respectively, complemented the uracil and uridine requirement of strain GR6. Transformants of the *A. oryzae *GR6 mutant carrying the heterologous expression vector ANEp2 were able to functionally express a secreted *β*-galactosidase at high levels.

## Supplementary Material

The cassette used for deleting the pyrG gene was 3,568 bp long. Boxed and lowercase letters indicated the XhoI site which fused the pyrG 5′ and 3′ UTR, respectively.
Multiple alignment of the (a) pyrG 5′ and (b) pyrG 3′ UTR sequence between *A. oryzae* RIB40 and *A. oryzae* strain S1, respectively. The alignment was constructed using the ClustalW (http://www.ebi.ac.uk/clustalw/) and BOXSHADE (http://www.ch.embnet.org/software/BOX_form.html) software

## Figures and Tables

**Table 1:** Primers used in this study.

Designation	Primer description	Primer sequence
5′pyr	*pyrG* 5′ region sense primer	5′-GGCGCCACAATCGTTGAGGTA-3′
*revint-XhoI	*pyrG* 5′ region antisense primer, the XhoI site is underlined	5′-CTCGAGGTTGGCGATGGAGG-3′
*fwdint-XhoI	*pyrG* 3′ region sense primer, the XhoI site is underlined	5′-CTCGAGGTAGTGGTGGATACGTACT-3′
3′pyr	*pyrG* 3′ region antisense primer	5′-AAGCTTATCAGCTGCATATCTCTAT-3′
Pyr5′-out	*pyrG* 5′ region sense primer outside the *pyrG* 5′ gene replacement cassette	5′-ATTTAGTCATTCCTTTTGGCATC-3′
Pyr3′-out	*pyrG* 3′ region antisense primer outside the *pyrG* 3′ gene replacement cassette	5′-CTAATTTTAATGTCTTCCTTCAG-3′

*revint-XhoI was used to amplify the targeting region downstream of the *pyrG* coding region.

*fwdint-XhoI was used to amplify the targeting region upstream of the *pyrG* coding region.
