# Supplementary material for: Development of a pyrG Mutant of Aspergillus oryzae Strain S1 as a Host for the Production of Heterologous Proteins
Source: ScientificWorldJournal. 2013 Nov 30;2013:634317. doi: 10.1155/2013/634317 (PMC3864154; doi:10.1155/2013/634317)
Supplement: Supplementary file 1 — The cassette used for deleting the pyrG gene was 3,568 bp long. Boxed and lowercase letters indicated the XhoI site which fused the pyrG 5′ and 3′ UTR, respectively. Multiple alignment of the (a) pyrG 5′ and (b) pyrG 3′ UTR sequence between A. oryzae RIB40 and A. oryzae strain S1, respectively. The alignment was constructed using the ClustalW (http://www.ebi.ac.uk/clustalw/) and BOXSHADE (http://www.ch.embnet.org/software/BOX_form.html) software [file 634317.f1.doc]

The supplementary data showing sequencing results confirming construct are shown below and will be provided to the journal as supplementary Figure 1.

GGCGCCACAATCGTTGAGGTAAGAATGATCTGCCAATATGTGAGCGAGTGCGGCTGACAACTATGATAGT 70

TTGGTAATACAACATCTCACGAATCGTACCTTGCATGTGCACTAAAGTTTAGTCAGATGTACAGTTAACA 140

AGAGATCTCGTGCCCGTCATATACCATGACTTTTCTCTGAGCGAGTCAGGGACTGATATCCCTATTCATG 210

ACTTAAATCTTGATCAGGTCAGTATCTTTGAGATTGATTTGAACTGATTCTCACCCATCAAAGTTCATGC 280

ACGCAAGCAATATCCAGTCGCCACGAGGGGATCCCGTATCTGTGCTTGGGAAAGCGAATGCACAGCCTAT 350

ATCCACTCAGGTTACTTCGACTAAGCCACGATCTCGATCATTAACTAAAGACCACGAGAGGGGAACTAGG 420

GAAATCCGTGATAGAATGAAGTATACAGTTGATTTTGTCAGCAAGGGATTCAAACCTAATACCAGAGGTG 490

ACTTTATCCAAGATTCCTTCACGACACTGGAAGAACTGCTTGAAGAGCTGCCTGAGTCAATCAGCTTCAA 560

TATCGAGATAAGTAGGTTATATGCTGCCACTGGTGGTTCTCCACTTGCGGGAGACAAAGCTAACAACGTC 630

CCAATGAAGAGTACCCCAGGCTTCATGAAGCTATAGAAGCAGGTGTAGCACCAGTGGCTATTGAAATCAA 700

CACCTTCATCGACAAAGCGCTTGAGAGACTCTTTTCTTACGGCAACAAAAAACGGACCATTATCCTATCC 770

5’ UTR

TCATTTACTCCCGAGATCTGCATTTTATTGGCCATCAAACAACAGACGTACCCTGTGATGTTCATCACTA 840

ATGCCGGCAAGCCTCCAGTTACGGATCGAGAGATGAGGGCTGCCAGCATACAGTCCGCTGTTCGATTTGC 910

CAAGAGGTGGAATTTATCTGGCCTTGTCTTTGCATCTGAGGCGCTGGTAATGTGCCCCAGGCTTGTCAGA 980

TATGTTCAACGATCAGGATTGATCTGTGGATCCTATGGATCTCAGAACAATATACCAGAAAATGCGAAGG 1050

TAAGTGCTTCTATATTGATCCTTAGTGCTTTCAAACTGTGATGTAGAAGTTGCTCGGTAGCTGATTAAAT 1120

ATTCTAGACCCAAGCCGCTGCTGGAATTGACATTATTATGGCCGATAGGGTTGGGCTTATTGCTATGTCC 1190

CTGAAAGGATATCAAAAGCAGGCAAAAAGCCAGGCATAATCCCCGCGTGGACGGTACCCTAAGGATAGGC 1260

CCTAATCTTATCTACATGTGACTGCATCGATGTGTTTGGTCAAAATGAGGCATGTGGCTCACCCCACAGG 1330

CGGAGAAACGTGTGGCTAGTGCATGACAGTCCCCTCCATAGATTCAATTTAATTTTTCGCGGCAATTGTC 1400

GTGCAGTTTGTATCTACATTTCATTCCATATATCAAGAGTTAGTAGTTGGACATCCTGATTATTTTGTCT 1470

AATTACTGAAAACTCGAAGTACTAACCTACTAATAAGCCAGTTTCAACCACTAAGTGCTCATTTATACAA 1540

TATTTGCAGAACCCCGCGCTACCCCTCCATCGCCAACctcgagGTAGTGGTGGATACGTACTCCTTTTAT 1610

GGCAGTATGTCGCAAGTATGATGCGATTTATAAATTCAGCACTCGAAATGACTACTACTATGTGTCTACG 1680

ACAGATACCCTCTCCGTACGAATAAGACACCTGCCTCGATATATGGACAAATTCAAAATCAGGGTCAAGG 1750

GTCATGTTTCAAAGTCACAACAATCTCCAACATAGACGAGAATTTGTACCGGAGTGTCTGAAGGTGCAGC 1820

TGGAGATTGGTCTATTTTCTTAGAGTGGGGTATCACTAATGTACAGTCGGTCACTATCGTACAAACAATC 1890

ACAATTATATACAAGATTTCCCACCACCCCCTACTCTAACACGGCACAATTATCCATCGAGTCAGAGCCT 1960

AGCCACCATTTGGTGCTCTCGTAGAGACCAAAGTATAATCCTGATCCGACAGCGGCCATAAACGTGTTGA 2030

TAGCACACCCTCGGAATAGTCCTCTCGGGCCATCTGTTCGTACAATCTCCCGTACGGTATTGATCATCCT 2100

TTTCTTCTGAGGTGCAGTTGTATCTGCAGCATCGAGCATGATTCGTGTCCGGACCATATCCATGGGTGCT 2170

GTCAAGACACTAGCTATACCGCCCGAGACCGCAGCACTTATTGCGGCTGTCGCTGCAGCCTCTCCGATTG 2240

TCGAATGGGCCTCTTTCTTTCCATACTCTCTTGGTCTTTCTAGCACCTTCTCTCGATCTCCGAATCTATA 2310

TTCAAAAATTCGATACCGAAAAGACTCGTACAGAGGCATCTGAATCGCCGACACTGGCAAGCTATGCGCC 2380

ACAAGAGCCGGGTATCCGCTCCAAAGCTGTCTAGGGTTGATAAACTTCTTGAAAGCTAGCCGTGTCGCTT 2450

TCTGGGCTACACCACCTACCCTTCCCCCAGCTACAGGTGCTGATGCGTCTGGATGGTGTGATTGGATCAT 2520

3’ UTR

CTGCGCGTTGTGTTTTAATGCATCAGCCGGAGCAAAGACTCCGCAAGCAGCAAGATCCGCAACGGAGGCT 2590

GCGCAAAAATCGGAAAAGAGCCGAGCTGAGCTAGACTCATGCGTTCCAAGTTTTTGATGTATGACTTGGA 2660

GTCCTGACTGTGCATACTCGTATGTGATGAAGAATGCGCCCGCTTTGACCCGTTATTTTTTGCTCAATGA 2730

ATATGAGTTGGGTAAGGATAATGTTGGGTATCACCAACCTGTGGGAAATGAGGCAGCGGTTACGCTCGCG 2800

ATACCTTGGTAAAGGCCGCGGAATATCCCTGGGTGTCTCCATATGCTGGTTCCTGTGTTGGTTCTCAGGA 2870

ACTGCGAATATTCGCGAGACTGAATGCGGGTTTTGATCGTATCTAAGGGATATGTGAATAGATCTAGTGA 2940

GAGGGATGCGGTTGTGCTTGCCTAGTTCCCTCAGGTTAATGTATGCTGTTTGTCCAAGGGGGTTAGAAAA 3010

TATACCAGCAATATACTAAGTGGCTCGGCGGCCATATTATTTGTCGATAGAGGCGGGAGGACGAGGATGT 3080

TCAATCGTGGATTTTGTAACCGTCCTGGCACACATCACCTTACAAGGCTTTGAGTTTTCGTTCTATAATT 3150

TGAGACAGGTCACAGTCGATTGCCATATTAGATTACAGCAACGTAGGTAGACATTAGGATTGATGCCAGG 3220

CAGCGAAGTTTTTTTCACTATTGGATGAGTTTATTCCGGGCTCCGCGGGAACCGATTGGGGTCTGACATT 3290

TCTGGAAAATCCCCAAACATATTTCATTTTGTCTTTGCGGACAGATAAAACGCTTTCGATTCCGATAGTA 3360

GAGGTATATACACATCGAGTGTAAACAGTATAAGCATATAGTCTCTCGGTTTGTTAGCGGTTTTGACGCA 3430

TACTAATAAATCATCGATAACTAGTACTGCTATCGATAAGCTCCTTGACGGGGAAAGGTAGCAGGACCCA 3500

CTGCAGTCACTTTAGTAAGTGGATGTCAGGATGATAAGGAGATATAGAGATATGCAGCTGATAAGCTT 3568

The cassette used for deleting the *pyrG* gene was 3,568 bp long. Boxed and lowercase letters indicated the *Xho*I site which fused the *pyrG* 5’ and 3’ UTR, respectively.

**a**

RIB40 1 GGCGCCACAATCGTTGAGGTAAGAATGATCTGCCAATATGTGAGCGAGTGCGGCTGACAA
S1 1 GGCGCCACAATCGTTGAGGTAAGAATGATCTGCCAATATGTGAGCGAGTGCGGCTGACAA
consensus 1 ************************************************************

RIB40 61 CTATGATAGTTTGGTAATACAACATCTCACGAATCGTACCTTGCATGTGCACTAAAGTTT
S1 61 CTATGATAGTTTGGTAATACAACATCTCACGAATCGTACCTTGCATGTGCACTAAAGTTT
consensus 61 ************************************************************

RIB40 121 AGTCAGATGTACAGTTAACAAGAGATCTCGTGCCCGTCATATACCATGACTTTTCTCTGA
S1 121 AGTCAGATGTACAGTTAACAAGAGATCTCGTGCCCGTCATATACCATGACTTTTCTCTGA
consensus 121 ************************************************************

RIB40 181 GCGAGTCAGGGACTGATATCCCTATTCATGACTTAAATCTTGATCAGGTCAGTATCTTTG
S1 181 GCGAGTCAGGGACTGATATCCCTATTCATGACTTAAATCTTGATCAGGTCAGTATCTTTG
consensus 181 ************************************************************

RIB40 241 AGATTGATTTGAACTGATTCTCACCCATCAAAGTTCATGCACGCAAGCAATATCCAGTCG
S1 241 AGATTGATTTGAACTGATTCTCACCCATCAAAGTTCATGCACGCAAGCAATATCCAGTCG
consensus 241 ************************************************************

RIB40 301 CCACGAGGGGATCCCGTATCTGTGCTTGGGAAAGCGAATGCACAGCCTATATCCACTCAG
S1 301 CCACGAGGGGATCCCGTATCTGTGCTTGGGAAAGCGAATGCACAGCCTATATCCACTCAG
consensus 301 ************************************************************

RIB40 361 GTTACTTCGACTAAGCCACGATCTCGATCATTAACTAAAGACCACGAGAGGGGAACTAGG
S1 361 GTTACTTCGACTAAGCCACGATCTCGATCATTAACTAAAGACCACGAGAGGGGAACTAGG
consensus 361 ************************************************************

RIB40 421 GAAATCCGTGATAGAATGAAGTATACAGTTGATTTTGTCAGCAAGGGATTCAAACCTAAT
S1 421 GAAATCCGTGATAGAATGAAGTATACAGTTGATTTTGTCAGCAAGGGATTCAAACCTAAT
consensus 421 ************************************************************

RIB40 481 ACCAGAGGTGACTTTATCCAAGATTCCTTCACGACACTGGAAGAACTGCTTGAAGAGCTG
S1 481 ACCAGAGGTGACTTTATCCAAGATTCCTTCACGACACTGGAAGAACTGCTTGAAGAGCTG
consensus 481 ************************************************************

RIB40 541 CCTGAGTCAATCAGCTTCAATATCGAGATAAGTAGGTTATATGCTGCCACTGGTGGTTCT
S1 541 CCTGAGTCAATCAGCTTCAATATCGAGATAAGTAGGTTATATGCTGCCACTGGTGGTTCT
consensus 541 ************************************************************

RIB40 601 CCACTTGCGGGAGACAAAGCTAACAACGTCCCAATGAAGAGTACCCCAGGCTTCATGAAG
S1 601 CCACTTGCGGGAGACAAAGCTAACAACGTCCCAATGAAGAGTACCCCAGGCTTCATGAAG
consensus 601 ************************************************************

RIB40 661 CTATAGAAGCAGGTGTAGCACCAGTGGCTATTGAAATCAACACCTTCATCGACAAAGCGC
S1 661 CTATAGAAGCAGGTGTAGCACCAGTGGCTATTGAAATCAACACCTTCATCGACAAAGCGC
consensus 661 ************************************************************

RIB40 721 TTGAGAGACTCTTTTCTTACGGCAACAAAAAACGGACCATTATCCTATCCTCATTTACTC
S1 721 TTGAGAGACTCTTTTCTTACGGCAACAAAAAACGGACCATTATCCTATCCTCATTTACTC
consensus 721 ************************************************************

RIB40 781 CCGAGATCTGCATTTTATTGGCCATCAAACAACAGACGTACCCTGTGATGTTCATCACTA
S1 781 CCGAGATCTGCATTTTATTGGCCATCAAACAACAGACGTACCCTGTGATGTTCATCACTA
consensus 781 ************************************************************

RIB40 841 ATGCCGGCAAGCCTCCAGTTACGGATCGAGAGATGAGGGCTGCCAGCATACAGTCCGCTG
S1 841 ATGCCGGCAAGCCTCCAGTTACGGATCGAGAGATGAGGGCTGCCAGCATACAGTCCGCTG
consensus 841 ************************************************************

RIB40 901 TTCGATTTGCCAAGAGGTGGAATTTATCTGGCCTTGTCTTTGCATCTGAGGCGCTGGTAA
S1 901 TTCGATTTGCCAAGAGGTGGAATTTATCTGGCCTTGTCTTTGCATCTGAGGCGCTGGTAA
consensus 901 ************************************************************

RIB40 961 TGTGCCCCAGGCTTGTCAGATATGTTCAACGATCAGGATTGATCTGTGGATCCTATGGAT
S1 961 TGTGCCCCAGGCTTGTCAGATATGTTCAACGATCAGGATTGATCTGTGGATCCTATGGAT
consensus 961 ************************************************************

RIB40 1021 CTCAGAACAATATACCAGAAAATGCGAAGGTAAGTGCTTCTATATTGATCCTTAGTGCTT
S1 1021 CTCAGAACAATATACCAGAAAATGCGAAGGTAAGTGCTTCTATATTGATCCTTAGTGCTT
consensus 1021 ************************************************************

RIB40 1081 TCAAACTGTGATGTAGAAGTTGCTCGGTAGCTGATTAAATATTCTAGACCCAAGCCGCTG
S1 1081 TCAAACTGTGATGTAGAAGTTGCTCGGTAGCTGATTAAATATTCTAGACCCAAGCCGCTG
consensus 1081 ************************************************************

RIB40 1141 CTGGAATTGACATTATTATGGCCGATAGGGTTGGGCTTATTGCTATGTCCCTGAAAGGAT
S1 1141 CTGGAATTGACATTATTATGGCCGATAGGGTTGGGCTTATTGCTATGTCCCTGAAAGGAT
consensus 1141 ************************************************************

RIB40 1201 ATCAAAAGCAGGCAAAAAGCCAGGCATAATCCCCGCGTGGACGGTACCCTAAGGATAGGC
S1 1201 ATCAAAAGCAGGCAAAAAGCCAGGCATAATCCCCGCGTGGACGGTACCCTAAGGATAGGC
consensus 1201 ************************************************************

RIB40 1261 CCTAATCTTATCTACATGTGACTGCATCGATGTGTTTGGTCAAAATGAGGCATGTGGCTC
S1 1261 CCTAATCTTATCTACATGTGACTGCATCGATGTGTTTGGTCAAAATGAGGCATGTGGCTC
consensus 1261 ************************************************************

RIB40 1321 ACCCCACAGGCGGAGAAACGTGTGGCTAGTGCATGACAGTCCCCTCCATAGATTCAATTT
S1 1321 ACCCCACAGGCGGAGAAACGTGTGGCTAGTGCATGACAGTCCCCTCCATAGATTCAATTT
consensus 1321 ************************************************************

RIB40 1381 AATTTTTCGCGGCAATTGTCGTGCAGTTTGTATCTACATTTCATTCCATATATCAAGAGT
S1 1381 AATTTTTCGCGGCAATTGTCGTGCAGTTTGTATCTACATTTCATTCCATATATCAAGAGT
consensus 1381 ************************************************************

RIB40 1441 TAGTAGTTGGACATCCTGATTATTTTGTCTAATTACTGAAAACTCGAAGTACTAACCTAC
S1 1441 TAGTAGTTGGACATCCTGATTATTTTGTCTAATTACTGAAAACTCGAAGTACTAACCTAC
consensus 1441 ************************************************************

RIB40 1501 TAATAAGCCAGTTTCAACCACTAAGTGCTCATTTATACAATATTTGCAGAACCCCGCGCT
S1 1501 TAATAAGCCAGTTTCAACCACTAAGTGCTCATTTATACAATATTTGCAGAACCCCGCGCT
consensus 1501 ************************************************************

RIB40 1561 ACCCCTCCATCGCCAAC
S1 1561 ACCCCTCCATCGCCAAC
consensus 1561 *****************

**b**

RIB40 1 GTAGTGGTGGATACGTACTCCTTTTATGGCAGTATGTCGCAAGTATGATGCGATTTATAA
S1 1 GTAGTGGTGGATACGTACTCCTTTTATGGCAGTATGTCGCAAGTATGATGCGATTTATAA
consensus 1 ************************************************************

RIB40 61 ATTCAGCACTCGAAATGACTACTACTATGTGTCTACGACAGATACCCTCTCCGTACGAAT
S1 61 ATTCAGCACTCGAAATGACTACTACTATGTGTCTACGACAGATACCCTCTCCGTACGAAT
consensus 61 ************************************************************

RIB40 121 AAGACACCTGCCTCGATATATGGACAAATTCAAAATCAGGGTCAAGGGTCATGTTTCAAA
S1 121 AAGACACCTGCCTCGATATATGGACAAATTCAAAATCAGGGTCAAGGGTCATGTTTCAAA
consensus 121 ************************************************************

RIB40 181 GTCACAACAATCTCCAACATAGACGAGAATTTGTACCGGAGTGTCTGAAGGTGCAGCTGG
S1 181 GTCACAACAATCTCCAACATAGACGAGAATTTGTACCGGAGTGTCTGAAGGTGCAGCTGG
consensus 181 ************************************************************

RIB40 241 AGATTGGTCTATTTTCTTAGAGTGGGGTATCACTAATGTACAGTCGGTCACTATCGTACA
S1 241 AGATTGGTCTATTTTCTTAGAGTGGGGTATCACTAATGTACAGTCGGTCACTATCGTACA
consensus 241 ************************************************************

RIB40 301 AACAATCACAATTATATACAAGATTTCCCACCACCCCCTACTCTAACACGGCACAATTAT
S1 301 AACAATCACAATTATATACAAGATTTCCCACCACCCCCTACTCTAACACGGCACAATTAT
consensus 301 ************************************************************

RIB40 361 CCATCGAGTCAGAGCCTAGCCACCATTTGGTGCTCTCGTAGAGACCAAAGTATAATCCTG
S1 361 CCATCGAGTCAGAGCCTAGCCACCATTTGGTGCTCTCGTAGAGACCAAAGTATAATCCTG
consensus 361 ************************************************************

RIB40 421 ATCCGACAGCGGCCATAAACGTGTTGATAGCACACCCTCGGAATAGTCCTCTCGGGCCAT
S1 421 ATCCGACAGCGGCCATAAACGTGTTGATAGCACACCCTCGGAATAGTCCTCTCGGGCCAT
consensus 421 ************************************************************

RIB40 481 CTGTTCGTACAATCTCCCGTACGGTATTGATCATCCTTTTCTTCTGAGGTGCAGTTGTAT
S1 481 CTGTTCGTACAATCTCCCGTACGGTATTGATCATCCTTTTCTTCTGAGGTGCAGTTGTAT
consensus 481 ************************************************************

RIB40 541 CTGCAGCATCGAGCATGATTCGTGTCCGGACCATATCCATGGGTGCTGTCAAGACACTAG
S1 541 CTGCAGCATCGAGCATGATTCGTGTCCGGACCATATCCATGGGTGCTGTCAAGACACTAG
consensus 541 ************************************************************

RIB40 601 CTATACCGCCCGAGACCGCAGCACTTATTGCGGCTGTCGCTGCAGCCTCTCCGATTGTCG
S1 601 CTATACCGCCCGAGACCGCAGCACTTATTGCGGCTGTCGCTGCAGCCTCTCCGATTGTCG
consensus 601 ************************************************************

RIB40 661 AATGGGCCTCTTTCTTTCCATACTCTCTTGGTCTTTCTAGCACCTTCTCTCGATCTCCGA
S1 661 AATGGGCCTCTTTCTTTCCATACTCTCTTGGTCTTTCTAGCACCTTCTCTCGATCTCCGA
consensus 661 ************************************************************

RIB40 721 ATCTATATTCAAAAATTCGATACCGAAAAGACTCGTACAGAGGCATCTGAATCGCCGACA
S1 721 ATCTATATTCAAAAATTCGATACCGAAAAGACTCGTACAGAGGCATCTGAATCGCCGACA
consensus 721 ************************************************************

RIB40 781 CTGGCAAGCTATGCGCCACAAGAGCCGGGTATCCGCTCCAAAGCTGTCTAGGGTTGATAA
S1 781 CTGGCAAGCTATGCGCCACAAGAGCCGGGTATCCGCTCCAAAGCTGTCTAGGGTTGATAA
consensus 781 ************************************************************

RIB40 841 ACTTCTTGAAAGCTAGCCGTGTCGCTTTCTGGGCTACACCACCTACCCTTCCCCCAGCTA
S1 841 ACTTCTTGAAAGCTAGCCGTGTCGCTTTCTGGGCTACACCACCTACCCTTCCCCCAGCTA
consensus 841 ************************************************************

RIB40 901 CAGGTGCTGATGCGTCTGGATGGTGTGATTGGATCATCTGCGCGTTGTGTTTTAATGCAT
S1 901 CAGGTGCTGATGCGTCTGGATGGTGTGATTGGATCATCTGCGCGTTGTGTTTTAATGCAT
consensus 901 ************************************************************

RIB40 961 CAGCCGGAGCAAAGACTCCGCAAGCAGCAAGATCCGCAACGGAGGCTGCGCAAAAATCGG
S1 961 CAGCCGGAGCAAAGATTCCGCAAGCAGCAAGATCCGCAACGGAGGCTGCGCAAAAATCGG
consensus 961 *************** ********************************************

RIB40 1021 AAAAGAGCCGAGCTGAGCTAGACTCATGCGTTCCAAGTTTTTGATGTATGACTTGGAGTC
S1 1021 AAAAGAGCCGAGCTGAGCTAGACTCATGCGTTCCAAGTTTTTGATGTATGACTTGGAGTC
consensus 1021 ************************************************************

RIB40 1081 CTGACTGTGCATACTCGTATGTGATGAAGAATGCGCCCGCTTTGACCCGTTATTTTTTGC
S1 1081 CTGACTGTGCATACTCGTATGTGATGAAGAATGCGCCCGCTTTGACCCGTTATTTTTTGT
consensus 1081 ***********************************************************

RIB40 1141 TCAATGAATATGAGTTGGGTAAGGATAATGTTGGGTATCACCAACCTGTGGGAAATGAGG
S1 1141 TCAATGAATATGAGTTGGGTAAGGATAATGTTGGGTATCACCAACCTGTGGGAAATGAGG
consensus 1141 ************************************************************

RIB40 1201 CAGCGGTTACGCTCGCGATACCTTGGTAAAGGCCGCGGAATATCCCTGGGTGTCTCCATA
S1 1201 CAGCGGTTACGCTCGCGATCCCTTGGTAAAGGCCGCGGAATATCCCTGGGTGTCTCCATA
consensus 1201 ******************* ****************************************

RIB40 1261 TGCTGGTTCCTGTGTTGGTTCTCAGGAACTGCGAATATTCGCGAGACTGAATGCGGGTTT
S1 1261 TGCTGGTTCCTGGGTTGGTTCTCAGGAACTGCGAATATTCGCGAGACTGAATGCGGGTTT
consensus 1261 ************ ***********************************************

RIB40 1321 TGATCGTATCTAAGGGATATGTGAATAGATCTAGTGAGAGGGATGCGGTTGTGCTTGCCT
S1 1321 TGATCGTATCTAAGGGATATGTGAATAGATCTAGTGAGAGGGATGCGGTTGTGCTTGCCT
consensus 1321 ************************************************************

RIB40 1381 AGTTCCCTCAGGTTAATGTATGCTGTTTGTCCAAGGGGGTTAGAAAATATACCAGCAATA
S1 1381 AGTTCCCTCAGGTTAATGTATGCTGTTTGTCCAAGGGGGTTAGAAAATATACCAGCAATA
consensus 1381 ************************************************************

RIB40 1441 TACTAAGTGGCTCGGCGGCCATATTATTTGTCGATAGAGGCGGGAGGACGAGGATGTTCA
S1 1441 TACTAAGTGGCTCGGCGGCCATATTATTTGTCGATAGAGGCGGGAGGACGAGGATGTTCA
consensus 1441 ************************************************************

RIB40 1501 ATCGTGGATTTTGTAACCGTCCTGGCACACATCACCTTACAAGGCTTTGAGTTTTCGTTC
S1 1501 ATCGTGGATTTTGTAACCGTCCTGGCACACATCACCTTACAAGGCTTTGAGTTTTCGTTC
consensus 1501 ************************************************************

RIB40 1561 TATAATTTGAGACAGGTCACAGTCGATTGCCATATTAGATTACAGCAACGTAGGTAGACA
S1 1561 TATAATTTGAGACAGGTCACAGTCGATTGCCATATTAGATTACAGCAACGTAGGTAGACA
consensus 1561 ************************************************************

RIB40 1621 TTAGGATTGATGCCAGGCAGCGAAGTTTTTTTCACTATTGGATGAGTTTATTCCGGGCTC
S1 1621 TTAGGATTGATGCCAGGCAGCGAAGTTTTTTTCACTATTGGATGAGTTTATTCCGGGCTC
consensus 1621 ************************************************************

RIB40 1681 CGCGGGAACCGATTGGGGTCTGACATTTCTGGAAAATCCCCAAACATATTTCATTTTGTC
S1 1681 CGCGGGAACCGATTGGGGTCTGACATTTCTGGAAAATCCCCAAACATATTTCATTTTGTC
consensus 1681 ************************************************************

RIB40 1741 TTTGCGGACAGATAAAACGCTTTCGATTCCGATAGTAGAGGTATATACACATCGAGTGTA
S1 1741 TTTGCGGACAGATAAAACGCTTTCGATTCCGATAGTAGAGGTATATACACATCGAGTGTA
consensus 1741 ************************************************************

RIB40 1801 AACAGTATAAGCATATAGTCTCTCGGTTTGTTAGCGGTTTTGACGCATACTAATAAATCA
S1 1801 AACAGTATAAGCATATAGTCTCTCGGTTTGTTAGCGGTTTTGACGCATACTAATAAATCA
consensus 1801 ************************************************************

RIB40 1861 TCGATAACTAGTACTGCTATCGATAAGCTCCTTGACGGGGAAAGGTAGCAGGACCCACTG
S1 1861 TCGATAACTAGTACTGCTATCGATAAGCTCCTTGACGGGGAAAGGTAGCAGGACCCACTG
consensus 1861 ************************************************************

RIB40 1921 CAGTCACTTTAGTAAGTGGATGTCAGGATGATAAGGAGATATAGAGATATGCAGCTGATA
S1 1921 CAGTCACTTTAGTAAGTGGATGTCAGGATGATAAGGAGATATAGAGATATGCAGCTGATA
consensus 1921 ************************************************************

RIB40 1981 AGCTT
S1 1981 AGCTT
consensus 1981 *****

Multiple alignment of the (a) *pyrG* 5’ and (b) *pyrG* 3’ UTR sequence between *A. oryzae* RIB40 and *A. oryzae* strain S1, respectively. The alignment was constructed using the ClustalW (http://www.ebi.ac.uk/clustalw/) and BOXSHADE (http://www.ch.embnet.org/software/BOX_form.html) software.
